# Application of quantitative real-time PCR for identifying respiratory tract pathogens in outpatients with RTIs

**DOI:** 10.3389/fpubh.2025.1531432

**Published:** 2025-04-29

**Authors:** Lin Qi, Yong Yang, Ziou Xu, Hongqiu Wang, Zhen Pan, Dan Zhao, Rui Liu, Haifang Zhang, Xiaofang Xie

**Affiliations:** ^1^Department of Clinical Laboratory, The Second Affiliated Hospital of Soochow University, Suzhou, China; ^2^MOE Key Laboratory of Geriatric Diseases and Immunology, Suzhou Medical College, Soochow University, Suzhou, China

**Keywords:** quantitative real-time PCR, respiratory tract infection, outpatients, infection status, respiratory tract pathogens

## Abstract

**Introduction:**

Respiratory tract infections (RTIs) caused by various pathogens, including viruses, bacteria, and fungi, pose significant public health challenges worldwide. Understanding the etiology and epidemiology of RTIs is necessary for clinical management, rational drug use, formulation of preventive measures, and vaccine development.

**Methods:**

Quantitative real-time PCR was used to detect and analyze respiratory pathogens in outpatients at a hospital in Suzhou, including FluA, FluB, RSV, ADV, HRV, MP, and SARS-CoV-2.

**Results:**

Among the 27,031 respiratory and throat swab samples, the positive rate of virus detection accounts for 25.6%. MP, SARS-CoV-2, and FluA, in particular, showed high positive rates among children, adolescents, and adults. The highest infection rates of RSV, HRV, and ADV were found in patients under 5 years old. High rates of FluA and FluB were observed in patients aged 5–17 and 18–44 years. However, the highest rate of SARS-CoV-2 infection was primarily observed in older adults. Seasonally, the infection rates of SARS-CoV-2 and FluA were highest in spring, FluB, RSV, and ADV in winter, HRV in autumn, and MP in summer and autumn.

**Conclusion:**

By analyzing the results of respiratory virus nucleic acid detection, we can gain a better understanding of the infection status of common respiratory viruses, providing a basis for clinical diagnosis and treatment.

## Introduction

Respiratory tract infections (RTIs) pose a significant threat to human health and contribute to high rates of illness and mortality worldwide (1). RTIs are common conditions affecting the respiratory system. Based on the site of infection, these diseases can be categorized into upper respiratory tract infections (URTIs) and lower respiratory tract infections (LRTIs), which are typically caused by bacteria, viruses, and atypical pathogens (2, 3). Viruses are responsible for a significant proportion of respiratory diseases based on factors such as declining herd immunity or the introduction of new viral subtypes into the population, which contribute to the periodic occurrence of epidemics (4, 5). The primary causes of acute respiratory infections are RNA and DNA viruses that are through air, characterized by rapidly transmission and high infectivity. The epidemic characteristics of RTIs vary across regions and are influenced by factors such as the environment, geography, and economy, and have a tendency to cause regional outbreaks.

Nevertheless, it is difficult to confirm the etiology of RTIs without pathogen nucleic acid tests. Viral culture used to be the gold standard for diagnosis, but it is a tedious and time-consuming procedure and does not apply to acute respiratory virus infection (6). Similarly, serology has limited capability for accurate diagnosis due to its low sensitivity (7). Meanwhile, although antigen-based assays, such as the indirect or direct immunofluorescence antibody assay, are widely used because of their ease of use, they have limited sensitivity and specificity (8). The nucleic acid amplification test (NAAT) has played an increasingly important role, because it yields immediate results and has high sensitivity and specificity (9). Thus, NAAT has become the new standard method for detecting respiratory viruses. Recently, PCR fluorescent probe methods have been developed to detect respiratory tract pathogens (RTPs).

Understanding the prevalence and transmission patterns of RTPs is crucial for rational and standardized use of drugs, the implementation of preventive measures, and the development of vaccines (10, 11). We summarize the results of RTP nucleic acid tests and some clinical data from outpatients in the region to understand the epidemic distribution of RTIs. Currently, RTPs in clinical practice were detected by six kits for nucleic acid detection of respiratory pathogens, including influenza virus A (FluA), influenza virus B (FluB), respiratory syncytial virus (RSV), human rhinovirus (HRV), adenovirus (ADV), *mycoplasma pneumoniae* (MP), and SARS-CoV-2 (12).

## Materials and methods

### Subjects

A total of 27,031 throat swab samples were included from outpatients from January 2023 to February 2024. The patients were categorized into the following age groups: infants (<5 years), adolescents (5–17 years), young adults (18–44 years), middle-aged adults (45–64 years), and older adults (≥65 years). The seasons were defined as spring (March to May), summer (June to August), autumn (September to November), and winter (December to February).

This study was approved by the Ethics Committee of The Second Affiliated Hospital of Soochow University (JD-LK-2020-041-02), and all participants provided written informed consent.

### Research methods

Viral nucleic acid was extracted from the collected throat swab samples according to the manufacturer’s instructions. Then, the PCR fluorescent probe amplification method was performed, and the results of respiratory pathogen detection were analyzed.

### Sample nucleate preparation

The collected throat swab samples were placed in the virus inactivation sampling tube (Zhejiang Gongdong Medical Technology, Z10001) (3 mL, Gongdong). After mixing by shaking, 300 μL of the sample was added to the Nucleic Acid Extraction Purification kit (64-T) (Sansure Biotech, 20210488) using the nucleic acid extraction instrument (Natch 32A), and the corresponding program was started. The extraction procedure was completed after approximately 15 to 30 min. The deep-well plate was then removed and the nucleic acids were transferred for further use.

### PCR amplification operation procedure

The Six Respiratory Pathogens Nucleic Acid Diagnostic Kit (PCR-Fluorescence Probing) (Sansure Biotech, 20213400256) comprised solution A, solution B, and enzyme mix. The total amplification system for each sample was 50 μL: 43.5 μL of PCR Mix A or B, 1.5 μL of enzyme mix, and 5 μL of the prepared test specimen. Then, the mixture was shaken and centrifuged, and the PCR reaction tubes were placed into the specimen wells of the automatic PCR analytical instrument for medical analysis (SLAN-96S, Shanghai Hongshi) for amplification. When the reactions were completed, the results were automatically saved, along with the numerical Ct value. The time cycle parameters set are shown below:

**Table:** 

No.	Step	Temperature	Time	Cycle No.
1	Reverse transcription	50°C	30 min	1
2	Pre-denaturation	95°C	1 min	1
3	Denaturation	95°C	15 s	45
	Annealing, extension, and fluorescence detection	60°C	30 s	
4	Device cooling (optional)	25°C	10 s	1

The SARS-CoV-2 and Influenza A/B Virus Multiplex Nucleic Acid Diagnostic Kit (PCR-Fluorescence Probing) (Sansure Biotech, 20213401060) comprised solution A, solution B, and enzyme mix. The total amplification system for each sample was 50 μL: 26 μL of SARS-CoV-2/Inf A/B-PCR Mix, 4 μL of enzyme mix, and 20 μL of the prepared test specimen. Then, the mixture was shaken and centrifuged, and the PCR reaction tubes were placed into the specimen wells of the automatic PCR analytical instrument for medical analysis (SLAN-96S, Shanghai Hongshi) for amplification. When the reactions were completed, the results were automatically saved, along with the numerical Ct value. The time cycle parameters set are shown below:

**Table:** 

No.	Step	Temperature	Time	Cycle No.
1	Reverse transcription	50°C	5 min	1
2	Pre-denaturation	95°C	1 min	1
3	Denaturation	95°C	10 s	41
	Annealing, extension, and fluorescence detection	60°C	20 s	

### Statistical analysis

Data analysis was performed using Prism 9.5 software. Infection rates among respiratory pathogens were compared using the chi-squared test. A probability (*P*) of less than 0.05 was considered statistically significant.

## Results

### Respiratory pathogen nucleic acid test result analysis

**Figure 1:**
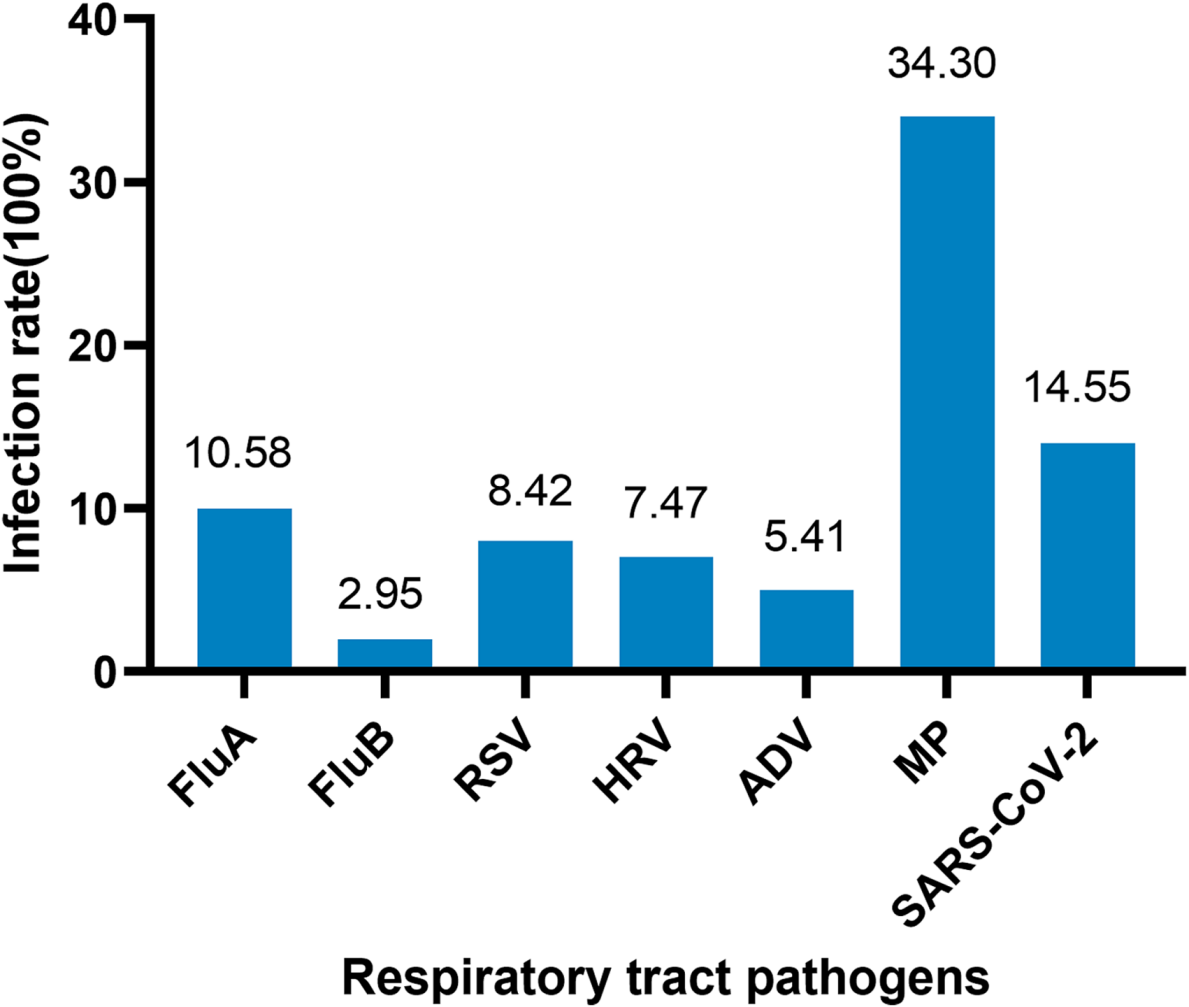
Single respiratory virus infection rate. Infants: <5 years; adolescents: 5–17 years; young adults: 18–44 years; middle-aged adults: 45–64 years; older adults: ≥65 years.

**Figure 2:**
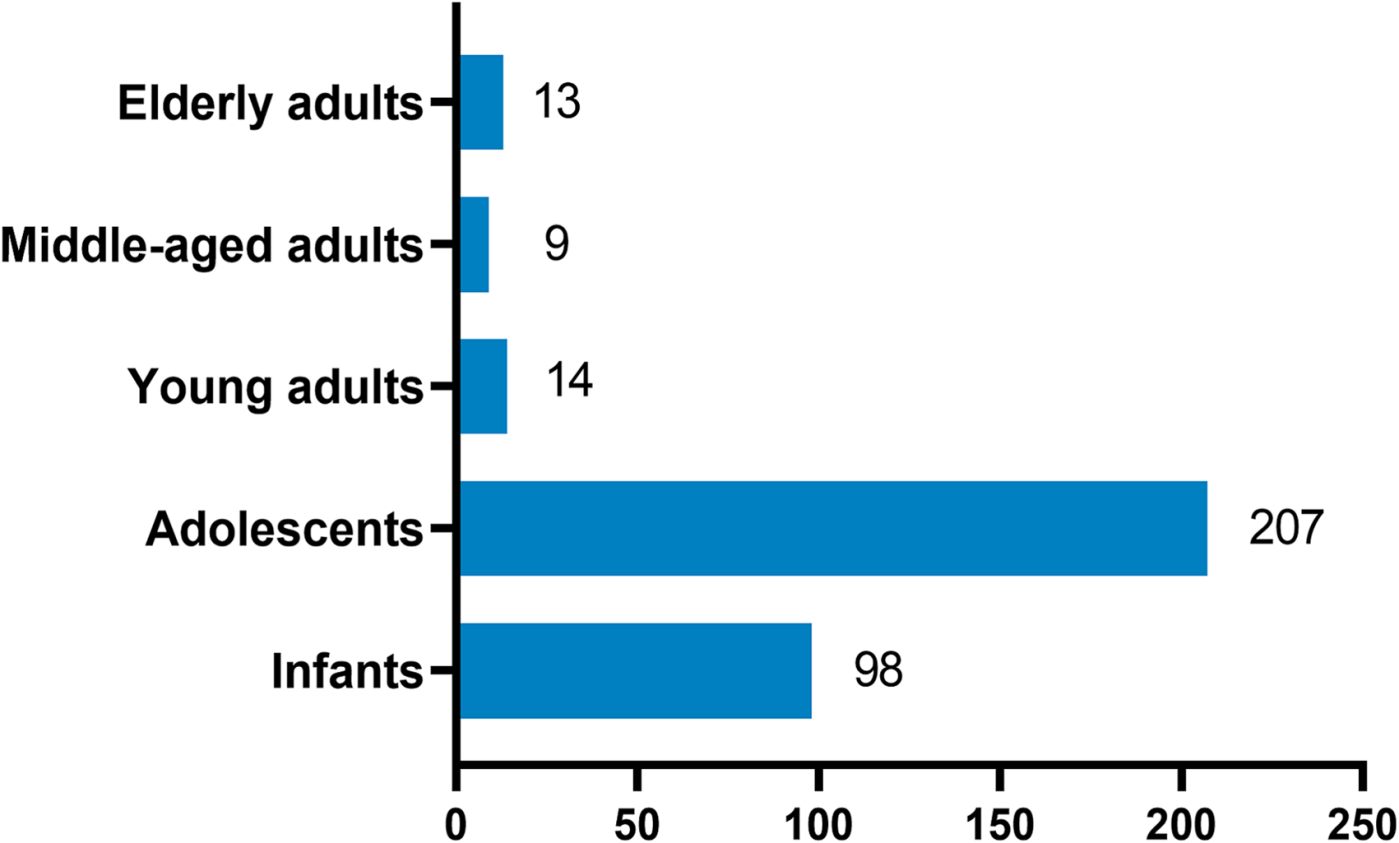
Mixed infection with more than two pathogens. FluA, influenza virus a; FluB, influenza virus b; RSV, respiratory syncytial virus; HRV, human rhinovirus; ADV, adenovirus; MP, *mycoplasma pneumoniae*; SARS-CoV-2, severe acute respiratory syndrome coronavirus 2. Positive rate (%) = positive cases/total cases (100%).

The study tested 27,031 throat swab samples of outpatients from almost all ages, and the total positive rate accounted for 25.6%. The first symptoms of outpatients were primarily abnormal cough, lung sounds, fever, and abnormal bronchial sounds. Information on the demographics and clinical characteristics of patients is shown in Table 1. A total of 6,919 patients (25.6%) tested positive for at least one pathogen, and 341 (1.26%) of these patients were co-infected with multiple pathogens, which were more common in infants and adolescents (Figure 1). The most common viruses were MP, SARS-CoV-2, and FluA, followed by RSV, HRV, and ADV (Figure 2). The least common etiological agent discovered in this study was FluB, which tested positive, and accounted for 3.4% (Table 1).

**Table 1:** Patient demographics and clinical characteristics.

Characteristics	*n* (%)
Pathogen infection rates (*n* = 27,031)	6,919 (25.60)
Co-infection of pathogens	341 (1.26)
Sex	
Male (*n* = 14,872)	3,724 (25.04)
Female (*n* = 12,159)	3,190 (26.24)
Age group	
Infants (<5 years) (*n* = 1,356)	822 (60.62)
Adolescents (5–17 years) (*n* = 2,423)	1,356 (55.96)
Young adults (18–44 years) (*n* = 6,089)	1,420 (23.32)
Middle-aged adults (45–64 years) (*n* = 5,525)	962 (17.41)
Older adults (≥65 years) (*n* = 11,586)	2,354 (20.32)
Clinical symptoms at presentation (*n* = 6,919)	
Abnormal bronchial sounds	1,245 (18.00)
Abnormal pulmonary symptoms	2,214 (32.00)
Cough	1,939 (28.00)
Fever	1,245 (18.00)
Pant	276 (4.00)

### Comparison of RTPI between both sexes

Overall, the total infection rate of RTPs in women was no different from that in men (26.24% vs. 25.04%). Among them, 14,872 were male and 12,159 were female (Table 1).

### Comparison of RTPI among age groups

There were significant differences in infection rates among various age groups. The total infection rate was highest in patients aged <5 years (60.62%) and 5–17 years (55.96%). The order of infection rates in all age groups was as follows: <5 years: MP > RSV > SARS-CoV-2 > HRV > FluA > ADV > FluB; 5–17 years: MP > SARS-CoV-2 > FluA> HRV > ADV > RSV > FluB; 18–44 years: MP > FluA > SARS-CoV-2 > FluB> HRV > ADV > RSV; 45–64 years: SARS-CoV-2 > MP > FluA > HRV > RSV > FluB > ADV; and ≥65 years: SARS-CoV-2 > FluA > MP ≈ RSV ≈ HRV > FluB > ADV. The highest infection rates for RSV, HRV, and ADV were found in patients aged <5 years. High rates of FluA and FluB were observed in the 5–17 and 18–44-year age groups. However, the highest rate of SARS-CoV-2 was observed in patients aged ≥65 years, followed by those aged <5 years (Table 2).

**Table 2:** Positive rate of each respiratory tract pathogen in all age groups.

Positive cases/total cases (100%)						
Respiratory tract pathogen	Infants	Adolescents	Young adults	Middle-aged adults	Older adults	*p*
	(<5 years)	(5–17 years)	(18–44 years)	(45–64 years)	(≥65 years)	
Flu A	9.66 (124/1283)	13.01 (244/1876)	14.02 (374/2668)	8.61 (374/2668)	9.2 (471/5119)	<0.001
Flu B	3.9 (50/1283)	4.96 (93/1876)	4.95 (132/2668)	2.27 (55/2668)	1.21 (62/5119)	<0.001
RSA	20.51 (249/1214)	5.39 (83/1541)	1.21 (5/412)	2.28 (9/394)	4.25 (47/1106)	<0.001
HRV	10.71 (130/1214)	8.89 (137/1541)	4.61 (19/412)	4.82 (19/394)	4.07 (45/1106)	<0.001
ADV	8.48 (103/1214)	7.66 (118/1541)	2.91 (12/412)	1.52 (6/394)	1.18 (13/1106)	<0.001
MP	24.13 (306/1268)	64.56 (1,208/1871)	31.19 (136/436)	10.78 (44/408)	4.55 (52/1106)	<0.001
SARS-CoV-2	15.66 (13/83)	10.84 (63/581)	13.41 (755/5630)	12.35 (628/5085)	16.38 (1700/10379)	<0.001

FluA, influenza virus a; FluB, influenza virus b; RSV, respiratory syncytial virus; HRV, human rhinovirus; ADV, adenovirus; MP, *mycoplasma pneumoniae*; SARS-CoV-2, severe acute respiratory syndrome coronavirus 2.

### Comparison of RTPI among seasons

**Figure 3:**
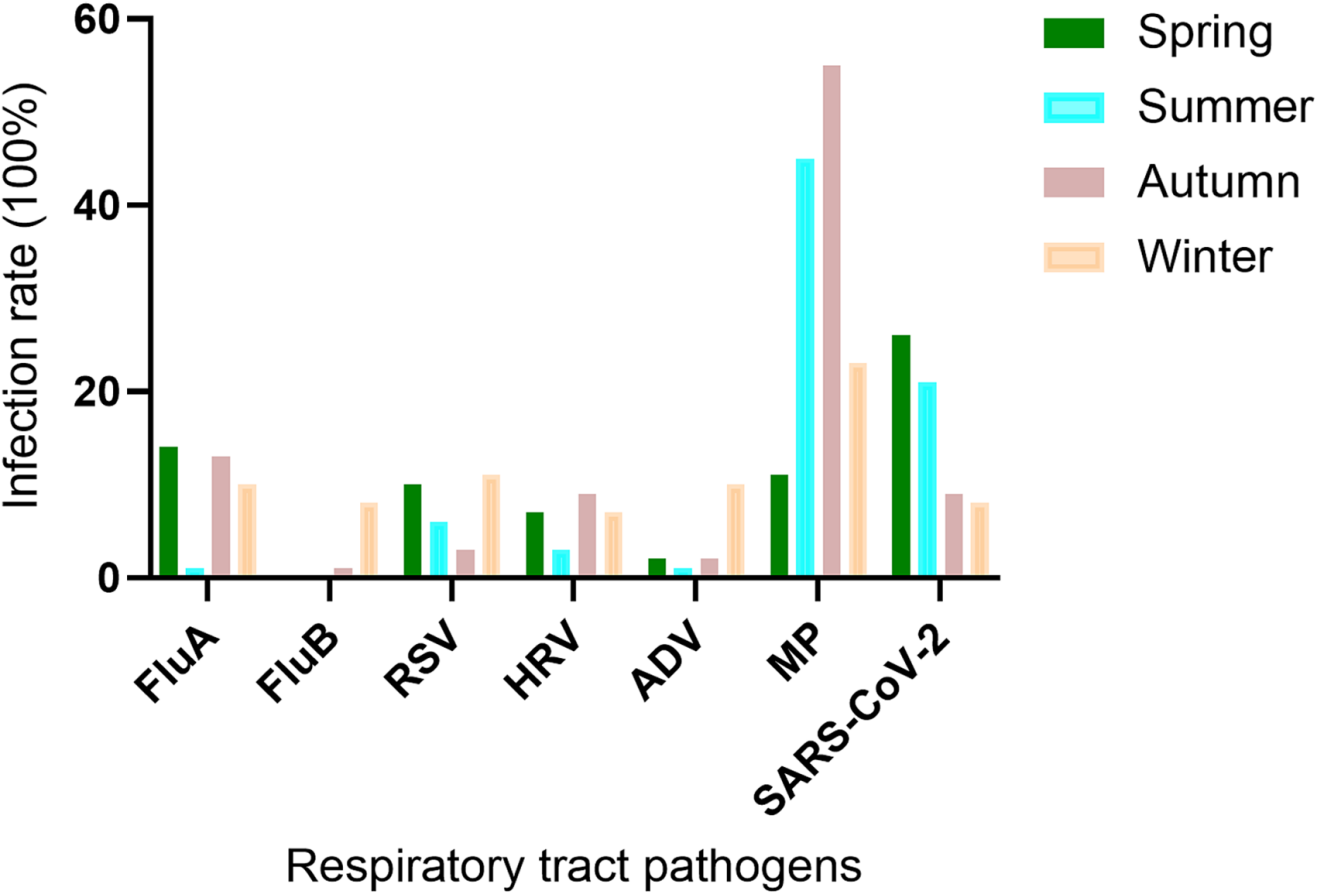
Seasonal distribution of RTPs in outpatients with RTs. FluA, influenza virus a; FluB, influenza virus b; RSV, respiratory syncytial virus; HRV, human rhinovirus; ADV, adenovirus; MP, *mycoplasma pneumoniae*; SARS-CoV-2, severe acute respiratory syndrome coronavirus 2. Positive rate (%) = positive cases/total cases (100%).

The study showed that RTPIs had clear seasonal variations. FluA was prevalent in spring, autumn, and winter, but its prevalence was very low in summer. FluB was primarily prevalent in winter, with very low occurrences in spring, summer, and autumn. The peak seasons for RSV infection were spring and winter. HRV was mainly prevalent in spring, autumn, and winter. ADV was most prevalent in winter, with an infection rate of 10.88%. The infection rate of MP remained high throughout all four seasons, while SARS-CoV-2 was primarily prevalent in spring and summer (Figure 3).

## Discussion

Various pathogens have been reported to cause emerging, re-emerging, and novel infectious diseases, leading to global epidemics. Viruses and some atypical pathogens (such as MP) can affect people of all ages, particularly vulnerable groups such as infants, older adults, patients with chronic diseases, and immunocompromised individuals (13). Viral RTIs typically present with symptoms such as cough, fever, fatigue, muscle pain, runny nose, and sweating (14). Our study also showed that the symptoms of patients at the time of presentation were mainly fever, cough, and pulmonary symptoms. Patients with viral pneumonia often exhibit the same symptoms as those with non-pneumonic viral infections, but the treatments are completely different (15). Therefore, physicians can use NAATs to ensure accurate diagnoses and reduce the unnecessary use of antibiotics. In addition, NAATs can act as a predictor of regional epidemics, and the corresponding prevention and control measures can be made in time.

The NAATs have reached a level of sensitivity, accuracy, and practicality that is routine for gene-level measurement in clinical laboratories (16, 17). Herein, a total of 6,919 throat swab samples (25.74%) were positive for a single pathogen, while the rate of mixed infections with multiple pathogens was 1.26%. MP and SARS-CoV-2 were the most commonly detected pathogens, followed by FluA, RSV, and HRV. The infection rate of FluB was only 2.75%, and it mainly occurred in winter.

The results of the data analysis revealed that infants, young children, older adults, and patients with chronic diseases are particularly susceptible to the virus. This finding is most likely due to reduced immune function or weaker resistance in these groups (14). Additionally, factors such as season and age contribute to differences in the groups infected with various respiratory pathogen infections (18, 19).

According to statistical analysis, FluA infections primarily occurred in young adults aged 18–44 years and in children and adolescents aged 5–17 years. The seasonal pattern of FluA had a particular peak in spring. Moreover, there were notable differences in the distribution of FluA and FluB infections across different age groups and seasons. In the region, the infection rate of FluA was much higher than that of FluB. Epidemiological studies have shown that seasonal climate changes and temperature variations are significantly correlated with virus survival and transmission rates (20, 21).

Based on the detection results of RSV, HRV, and ADV, infections can occur in any age group. RSV is primarily prevalent in spring and winter, with the highest infection risk observed among infants under 5 years old. However, certain groups, such as older adults, infants under 6 months of age, and individuals with compromised immune systems, may develop severe illness requiring further treatment, such as acetaminophen for fever reduction and salbutamol for wheezing relief (22). HRV is prevalent in autumn, winter, and spring, with infants under 5 years old and some children and adolescents being the most susceptible. This poses a significant health risk to infants and young children. The virus is currently believed to contribute to the worsening of chronic lung diseases, such as asthma, bronchiectasis, COPD, and bronchiolitis, which are among the leading causes of morbidity and mortality among older adults worldwide (23). Additionally, ADV infections mainly occur in winter, affecting infants, young children, and some adolescents, particularly those with low immune function. ADV is more likely to exacerbate illness and even increase the risk of death. Currently, there is no specific antiviral drug for adenovirus infection, so most treatments focus on symptom relief (24, 25).

The analysis showed that SARS-CoV-2 had a high infection rate across all age groups, with an overall positive rate of 14.52%, which is primarily prevalent in spring and summer. It can be inferred that, since the outbreak of SARS-CoV-2 in 2019, changes in the epidemic situation and adjustments in prevention and control strategies have led to a decline in the spread of the virus. Individuals with natural immunity or those who have been vaccinated may have developed immunity, which is a major factor in the decline of the SARS-CoV-2 epidemic (26). While there was no significant difference in SARS-CoV-2 infection rates between different age groups, the infection rate was higher among individuals over 65 years old than in other age groups. This variability may be due to the gradual decline in immune function with age, making them a group that is particularly susceptible to SARS-CoV-2 infection.

MP is the smallest prokaryotic pathogenic microorganism that is capable of invading not only the human respiratory system but also the cardiovascular, digestive, and nervous systems (27). When MP infection is confirmed, the primary treatment method is drug therapy, with macrolide antibiotics such as azithromycin, clarithromycin, and roxithromycin being the first choice (28). Proper use of antibiotics can alleviate symptoms and shorten the duration of the disease. MP is more common in school-age children over 5 years old; imaging typically shows bronchitis or bronchopneumonia (29). Therefore, precise diagnosis of pathogens in a timely manner can effectively guide clinical treatment and prevent antibiotic misuse. According to respiratory pathogen detection results in our hospital, MP infection accounted for 34.07% of single pathogen infections and was prevalent year-round. MP infection rates among male and female participants were equal, with the highest infection rates occurring in the 5 to 17-year-old age group, particularly in summer and autumn, which warrants close attention.

From the results of the analysis of local respiratory pathogens tested by quantitative real-time PCR, we can understand the epidemic characteristics of pathogens. This reminds us of the importance of non-drug preventive measures and the need to improve immunity. After infection, accurate diagnosis and standardized treatment are crucial. Furthermore, these measures can reduce the economic burden on patients and society and make effective use of healthcare resources.

## Conclusion

The landscape of respiratory infections is complex and continuously evolving, influenced by factors such as pathogen mutation, population immunity, and environmental changes. Ongoing research and public health efforts are essential to better understand these infections, develop precision diagnostic and treatment strategies, and implement effective prevention measures.

## Data Availability

The original contributions presented in the study are included in the article/supplementary material, further inquiries can be directed to the corresponding author.
